# Epidemic Impacts of a Community Empowerment Intervention for HIV Prevention among Female Sex Workers in Generalized and Concentrated Epidemics

**DOI:** 10.1371/journal.pone.0088047

**Published:** 2014-02-06

**Authors:** Andrea L. Wirtz, Carel Pretorius, Chris Beyrer, Stefan Baral, Michele R. Decker, Susan G. Sherman, Michael Sweat, Tonia Poteat, Jennifer Butler, Robert Oelrichs, Iris Semini, Deanna Kerrigan

**Affiliations:** 1 Center for Public Health and Human Rights, Department of Epidemiology, The Johns Hopkins Bloomberg School of Public Health, Baltimore, Maryland, United States of America; 2 Department of Emergency Medicine, Johns Hopkins Medical Institute, Baltimore, Maryland, United States of America; 3 Futures Institute, Glastonbury, Connecticut, United States of America; 4 Department of Population, Family, and Reproductive Health, The Johns Hopkins Bloomberg School of Public Health, Baltimore, Maryland, United States of America; 5 Medical University of South Carolina, Charleston, South Carolina, United States of America; 6 HIV and Key Populations, United Nations Population Fund, New York, New York, United States of America; 7 Human Development Network, The World Bank, Washington, District of Columbia, United States of America; 8 Department of Health, Behavior & Society, The Johns Hopkins Bloomberg School of Public Health, Baltimore, Maryland, United States of America; Yale School of Public Health, United States of America

## Abstract

**Introduction:**

Sex workers have endured a high burden of HIV infection in and across HIV epidemics. A comprehensive, community empowerment-based HIV prevention intervention emphasizes sex worker organization and mobilization to address HIV risk and often includes community-led peer education, condom distribution, and other activities. Meta-analysis of such interventions suggests a potential 51% reduction in inconsistent condom use. Mathematical modeling exercises provide theoretical insight into potential impacts of the intervention on HIV incidence and burden in settings where interventions have not yet been implemented.

**Methods:**

We used a deterministic model, Goals, to project the impact on HIV infections when the community empowerment interventions were scaled up among female sex workers in Kenya, Thailand, Brazil, and Ukraine. Modeling scenarios included expansion of the comprehensive community empowerment-based HIV prevention intervention from baseline coverage over a 5-year period (5–65% in Kenya and Ukraine; 10–70% in Thailand and Brazil), while other interventions were held at baseline levels. A second exercise increased the intervention coverage simultaneously with equitable access to ART for sex workers. Impacts on HIV outcomes among sex workers and adults are observed from 2012–2016 and, compared to status quo when all interventions are held constant.

**Results:**

Optimistic but feasible coverage (65%–70%) of the intervention demonstrated a range of impacts on HIV: 220 infections averted over 5 yrs. among sex workers in Thailand, 1,830 in Brazil, 2,220 in Ukraine, and 10,800 infections in Kenya. Impacts of the intervention for female sex workers extend to the adult population, cumulatively averting 730 infections in Thailand to 20,700 adult infections in Kenya. Impacts vary by country, influenced by HIV prevalence in risk groups, risk behaviors, intervention use, and population size.

**Discussion:**

A community empowerment approach to HIV prevention and access to universal ART for female sex workers is a promising human rights-based solution to overcoming the persistent burden of HIV among female sex workers across epidemic settings.

## Introduction

Female sex workers have endured a high burden of HIV infection in and across concentrated and generalized HIV epidemics. A recent meta-analysis calculated a pooled global HIV prevalence of 11.8% among female sex workers, estimating that, globally, female sex workers are 14 times more likely to be infected with HIV than adult women. Stratified by epidemic state, countries with medium or high prevalence among the adult population had an even greater pooled prevalence - as high as 30.7% across these countries. [1] A recent global analysis supports these findings, estimating that 15% of HIV infection in the general population is attributable to unsafe sex in the context of female sex work. Sub Saharan Africa contributes the greatest burden of HIV infection and, with respect to sex work, is estimated to contribute 98,000 of the global 106,000 HIV-related deaths attributable to sex work (approximately 92%), followed by high burden regions in Latin America and the Caribbean and South and South East Asia. [2] Though individual exposure during sex work may explain the high burden of HIV among sex workers, social and structural factors, which are highly variable across settings, can challenge or facilitate availability, access to, and uptake of HIV prevention interventions among women who sell sex.

Globally, a range of HIV prevention intervention strategies have been implemented in the context of sex work and have achieved varying degrees of success. A recent review by Cherish and colleagues of HIV prevention interventions for female sex workers in Sub-Saharan countries, suggested there was evidence of behavioral interventions to effectively reduce unprotected sex. [3] Peer-driven condom promotion and risk reduction counseling demonstrated effectiveness, as did combinations of clinical and peer-based services.[3]–[10] STI screening and presumptive treatment allowed for identification of and treatment for infection, though high coverage of return visits for STI care and treatment was not well maintained in some settings.[5], [11]–[13] Contrary to recent recommendations by the WHO, interventions that are currently considered priority for female sex workers –HIV counseling and testing, access to ART, and structural interventions - have rarely been evaluated or reported in the Sub-Saharan region. [3].

While stand-alone interventions are pertinent to preventing HIV transmission and acquisition, the high prevalence of HIV among female sex workers that is observed globally suggests the traditional stand-alone interventions are insufficient. [1] Combination or comprehensive approaches are now priority methods for key populations at risk for HIV infection. Combination approaches target prevention at a variety of potential transmission and acquisition opportunities through the use of effective behavioral, biomedical, and structural interventions. [14].

Combination HIV prevention has also been supported by recent research of contextual factors that may potentiate vulnerability to HIV infection for female sex workers. [15] Contextual factors include both macro and micro-level factors in the social, physical, structural, and economic environments that shape individual risk for HIV infection. Commonly described contextual factors include the regulation of sex work and economic opportunities, while other factors such as safe spaces for sex work, relationship dynamics, and many others create situations for protection against or vulnerability to HIV and other health risks. For female sex workers, combination approaches that consider these broader contextual factors and include community involvement in the design and implementation of the intervention have had successful outcomes in several settings and may have successful outcomes in future programs. [15].

In light of the structural factors that influence HIV vulnerabilities, a community empowerment-based HIV prevention intervention for female sex workers has been proposed as a promising method to address the range of individual and contextual factors that influence vulnerability to HIV infection. [16], [17] This approach to HIV prevention includes sex worker organization, mobilization, and collective action to address social and structural factors related to sex worker rights, health and HIV risk. It also considers more traditional programmatic elements, including community-led peer education, condom distribution, and STI/HIV screening and treatment. [16] A recent meta-analysis of 10 studies from low and middle income countries where community empowerment interventions were implemented found protective effects associated with intervention use. [18] These effects included reductions in STI, such as reduced gonorrhea/chlamydia observed in a longitudinal study (OR: 0.51; 95%CI: 0.26–0.99) and reduced gonorrhea in observational studies (OR: 0.65: 95%CI: 0.47–0.90). [18] Improvements in condom use with clients were observed in a randomized controlled trial (RCT; β: 0.3447; p = 0.002), with regular clients in a longitudinal study (OR: 1.9; 95%CI: 1.1–3.3), and across various client types (new, regular, and all) in observational studies, with pooled ORs ranging from 2.20 to 5.87. [18] Finally, HIV infection outcomes from observational studies were evaluated, demonstrating a significant combined protective effect associated with the intervention (OR: 0.84, 95%CI: 0.71–0.99). [18] Recently, guidance provided by the WHO, UNFPA, and UNAIDS and community consultation with the Network of Sex Work Project (NSWP) has affirmed these findings giving strong recommendations for a comprehensive, community empowerment-based HIV prevention intervention, though highlighting the current paucity of data on the effect of the intervention across settings. [17].

Recognizing that the comprehensive, community empowerment-based has been tested only in a few settings, we conducted a modeling exercise to provide a theoretical estimate of how such an intervention may impact HIV epidemics across heterogeneous populations and epidemic contexts. We conducted mathematical modeling using the Goals model to estimate impacts of expanding the coverage of comprehensive, community empowerment-based HIV prevention and access to universal ART on the HIV epidemics among female sex workers and the overall adult population in four case study countries.

## Methods

We selected four countries Kenya, Thailand, Brazil, and Ukraine, to model the impacts of scaling up community empowerment-based HIV prevention among female sex workers. Recognizing the effect of ART on HIV transmission and ART expansion and coverage as a core component of national strategies, a second modeling approach was conducted to assess the impact of providing equal access to ART for sex workers, during universal expansion, and the combined impact when the empowerment-based intervention is simultaneously expanded. Universal expansion was based on each country’s national targets for ART expansion. The models were calibrated to their respective country epidemics, and were utilized to estimate the number of new HIV infections among female sex workers and the adult population when intervention coverage and combinations were varied. Countries were selected to represent geographic, political, social, and epidemic diversity, including both concentrated and generalized epidemics and some with significant injecting drug use. Specifically, sex work is criminalized in Kenya [19] and is an administrative offense in Ukraine; however, sex work and is not illegal in Brazil and Thailand, though certain aspects, such as brothel owning and pimping are illegal in all countries. [20], [21] In Brail, sex work is as an official occupation. [22], [23] All four countries identify sex workers as key populations in their national HIV strategies and reporting mechanisms.

### Model Description

The Goals projection model, developed by Futures Institute and often used by countries to estimate national prevalence of HIV infection and future incidence projections, was applied using the updated Spectrum 2011 suite (v. 4.14 Beta16) to selected case countries. The model is used to project HIV incidence and prevalence among the general adult population and female sex workers in low and middle income countries when the community empowerment intervention to prevent HIV transmission among sex workers is brought to scale and/or when ART increases in coverage among the eligible adult population. [24].

The full description of the Goals model [24], details of parameterization, and use of the Goals model to evaluate impacts of key population interventions are described elsewhere. [16], [25], [26] Briefly, the Goals model is a deterministic model, integrated within the Spectrum suite of policy tools that uses data in several key areas to project HIV prevalence and incidence: demography; sexual behavior; and HIV and sexually transmitted infection (STI) prevalence. The Goals model uses population level demographic and epidemic projections from other Spectrum modules, such as the AIDS Impact Model (AIM) and DemProj (demographic projection), to create a country baseline HIV model. AIM is used to provide statistical estimates of HIV incidence. We provided further estimates of risk group behavioral and epidemic parameters for each country. [24].

### Model Parameters

Individuals are categorized by population risk groups within Goals as low, medium and high-risk heterosexuals, injecting drug users, and men who have sex with men (MSM). Within Goals female sex workers pertain to the category ‘High Risk Female Heterosexual’ and male clients of female sex workers pertain to ‘High Risk Male Heterosexual’. Though female sex workers are not the same as ‘high risk women’, this is the model term and the category was parameterized specifically for female sex workers for this particular estimation. This analysis did not focus on other risk groups such as MSM, transgender men and women, people who inject drugs; however, these groups as well as low and medium risk populations are included in the model to ensure that the variety of behaviors and differential risks observed in each modeled country is properly assessed. We did not test any behavior change among MSM, transgender, or IDU, thus no model change is attributable to these groups. Female sex workers do interact with high risk males as well as a specified percentage of low risk males and also transition to the medium risk female category after the duration of sex work is complete; therefore, inclusion of these populations in the model is important to assessing adult outcomes.

We edited Goals models with population size estimates and behavioral parameters for risk group in each country model according to research or surveillance data that are available. Data were inputted for female sex workers, sex work clients, MSM, male and female people who inject drugs, and heterosexual risk populations. During model simulations, when average duration of sex work has been met (i.e. the average duration in which women have been documented to be involved in sex work in each country), the population is re-allocated to the ‘Medium Risk Female Heterosexual’ category. Data inputs for selected countries were derived from the most recent and quality data available from population studies, UNGASS or UNAIDs country reports, surveillance reports, and country expert opinion if data were unavailable. For female sex workers, inputs included behavioral parameters, such as duration of sex work, numbers of clients, marital status or steady partnership).[27]–[40] Table 1 depicts the model parameters for female sex workers per country.

**Table 1:** Key Epidemic and Behavioral Parameters.

	Brazil			Kenya				Thailand			Ukraine		
Parameter	Estimate	Range	Source(s)	Estimate		Range	Source(s)	Estimate	Range	Source(s)	Estimate	Range	Source(s)
**Female sex worker population size**	0.9%	0.58–1.42%	[70]	4.3%		>100,000 or <6%	[47]	0.6%	150,000	[33], [71]	0.4%	65–95,000	[34], [49]
**Baseline adult HIV prevalence (2011)**	0.4%	0.6–0.7%	[53], [72]	6.0%		5.8–6.5%	[46], [47], [73]	1.0%	1.0–3.0%	[48], [52]	1.1	1.2–2.0%	[42], [49]
**Baseline prevalence of HIV among FSW (2011)**	4.9%	2.6–12.9%	[39], [72]	33.8%		3.4–66.8%	[31], [32], [74], [75]	5.0%	3.0–30.0%	[35], [36], [48]	13.2%	0–39%	[34], [49], [76]
**Prevalence of ulcerative STI among FSW (2011)**	17.0%	14.3–17.6%	[77],	20.0%		1.8–34.1%	[75], [79], [80]	10.0%	1.0–12.4%	[35], [81], [82]	13.0%	0–18.2%	[76]
**Consistent condom use among FSW**	75%	52.2–77.8%	[39], [70]	60%		29–91%	[45], [79], [83]–[85]	80%	78.9–96.0%	[86]	70%	66–86%	[42], [87]
**Number of clients per year**	205	40–140	[77]	90		50–180	[83], [84], [88], [89]	200	200–300	[71]	260	50–728	[38], [76], [87]
**Proportion of FSW married/in stable relationships**	20%	14.3–40.9%	[77], [78], [90]	45%		25.9–67.0%	[75], [83], [91]	35%	35.1%	[92]	4%	4–22%	[38], [87]
**Duration in sex work (years)**	8	5–10	[93], [94]	5		2–6	[95]	5	1–10	[81], [82]	10	0.5–15	[38]
**Intervention impacts (values for all country case studies)**													
**Impact of Empowerment Intervention on condom non-use**					−51.0%								[16], [17]
**Reduction in transmission on ART**					0.13								[55]
CD4 count at ARV initiation*					≤350 cells/mm^3^								[54]

Note: FSW: Female sex worker; *Criterion used for this analysis based on guidance at time of modeling analysis.

Epidemic parameters, including HIV and ulcerative STI (such as syphilis), were also edited for each risk group per country, according to published research and surveillance.[41]–[49] Baseline intervention coverage levels for the four countries were estimated based on country level reporting or expert input and presented as percentages. Adult and risk group epidemic curves were then fit and calibrated against UNAIDS projections for adult prevalence and fit to within 95% confidence intervals around the UNAIDS estimates.[39], [50]–[52] The female sex worker epidemic curves were calibrated to historical HIV epidemics among sex work populations estimated by surveillance reports. If surveillance estimates were unavailable, pooled estimates from epidemiologic research studies were utilized.

Goals projections are calculated based on these behavioral and epidemic data as well as intervention effectiveness and coverage. Changes in behavioral intervention coverage is mapped through an impact matrix to change in the behavior of those risk groups reached by the intervention. This ultimately changes risk behavior and the number of new infections per group and total population. Depending on intervention effect, the risk group reached, and associated behaviors some interventions may have wider impact than others.

### Model Scenarios and Analysis Plan

Two key interventions were the focus of the impact projections for these countries: comprehensive, community empowerment-based HIV prevention for sex workers and scale-up of universal ART for eligible adults living with HIV. Eligibility, ART initiation, and national coverage and expansion were based on national strategies for each specific country.[41], [42], [44]–[46], [48], [53] ART initiation for all adults was fixed at an eligibility of CD4≤350 cells/mm^3^, based on the national strategies and the revised WHO treatment guidelines, at the time of analysis. [54] Initiation of ART at CD4≤350 cells/mm^3^ was estimated to have a demonstrated effectiveness value of 88–96% reduction in risk associated with ART at this CD4 count, based on HPTN 052. [55] We used a slightly conservative estimate of 87% reduction in transmission when on ART. In the 2011 version of Goals, ART coverage can be scaled-up only among the total adult populations according to CD4 criterion and cannot be specifically brought to scale among specified risk groups, such as sex workers. As such, these estimates assume equitable access to ART for individuals from across groups and meeting CD4 eligibility requirements. Since FSW are typically marginalized with respect to ART access, this assumption is essentially equivalent to an intervention for FSW.

Behavioral intervention impacts are taken into consideration in the Goals model through the impact matrix. In this case, impacts associated with the community empowerment intervention among sex workers is assumed to be related to the reduction in inconsistent condom use. Another behavioral impact typically utilized to assess prevention of sexually transmitted/acquired HIV infection, is the reduction in number of sexual partners (or clients, in the case of sex workers). Given the occupational perspective of sex work, however, and that reduction in number of partners or clients would contradict a community empowerment approach for sex workers, this study did not investigate such a reduction in clients or partners and focused, instead, on reduction in condom nonuse. Thus, partner and client numbers that were inputted into the model parameters were held constant for all modeling scenarios.

The joint WHO/UNAIDS/UNFPA/NSWP systematic review of HIV interventions for sex workers [17] provided the impact matrix input value for the community empowerment intervention. Data from relevant publications were further meta-analyzed using data from all studies related to consistent condom use with all clients across study designs, which ultimately calculated a risk ratio in condom use of 1.77 (95%CI: 1.44, 2.203). [17] This value was recalculated using the formula for relative decrease in non-use: D = (RR-1) × (1-c_l_)/c_l_ in which c_l_ was the pooled condom use at intervention baseline. This calculation estimated a value of −51.0% reduction in condom non-use associated with the intervention (Table 1). Table 1 also depicts the modes of transmission, associated transmission efficiencies, and data sources used for this modeling exercise. The estimated baseline coverage of the comprehensive, community empowerment-based HIV prevention intervention for female sex workers was specified for each country according to published research or reports and expert opinion. [34], [38], [41], [45]–[48], [56].

### Modeling Scenarios and Analysis

We ran two sets of scenarios: the first focused on the comprehensive, community empowerment-based HIV prevention intervention for female sex workers. We assessed the impact of this intervention at varying coverage levels while holding ART coverage and coverage of all interventions among adults constant from the 2011 forward through 2016. The community empowerment intervention was increased from baseline coverage (5% coverage in Kenya and Ukraine and 10% in Thailand and Brazil) to reach the target coverage levels by 2016. We modeled several scenarios with different target coverage levels (Table 2), the highest coverage being 100%; however, we report in the text the results of the coverage that we believe to be both optimistic but feasible (65% in Kenya and Ukraine and 75% in Thailand and Brazil).

**Table 2:** Baseline and 2016 target coverage levels of the community empowerment-based prevention intervention and ART per scenario and country.

Modeling scenarios		Brazil	Kenya	Thailand	Ukraine
*Status quo*	*Baseline coverage of Empowerment Intervention held constant among female sex workers (2011*–*16)*	*10.0%*	*5.0%*	*10.0%*	*5.0%*
	ART coverage of adults (%) held constant among total population* (2011–*16)*	*66.2%*	*62.7%*	*67.0%*	*12.0%*
**Empowerment Intervention modeling scenarios**					
*Scenario 1:*	Interpolated scale-up of Empowerment coverage from 2011 to additional 30% by 2016	40.0%	35.0%	40.0%	35.0%
	ART coverage of adults (%) held constant among total population* (2011–*16)*	*66.2%*	*62.7%*	*67.0%*	*12.0%*
*Scenario 2:*	Interpolated scale-up of Empowerment coverage from 2011 to additional 60% by 2016	70.0%	65.0%	70.0%	65.0%
	ART coverage of adults (%) held constant among total population* (2011–*16)*	*66.2%*	*62.7%*	*67.0%*	*12.0%*
*Scenario 3:*	Interpolated scale-up of Empowerment coverage from 2011 to reach 100% by 2016	100.0%	100.0%	100.0%	100.0%
	ART coverage of adults (%) held constant among total population* (2011–*16)*	*66.2%*	*62.7%*	*67.0%*	*12.0%*
**ART modeling scenarios**		**Brazil**	**Kenya**	**Thailand**	**Ukraine**
*Scenario 1:*	Scale-up in coverage of ART by 2016 according to country estimations (% coverage)*	80.0%	85.0%	71.2%	24.0%
	*Baseline coverage of Empowerment Intervention held constant among female sex workers (%, 2011*–*16)*	*10.0%*	*5.0%*	*10.0%*	*5.0%*
*Scenario 2:*	Scale-up in coverage of ART among adults: % covered by 2016	80.0%	85.0%	71.2%	24.0%
	Interpolated scale-up of Empowerment coverage from 2011 to additional 60% by 2016	70.0%	65.0%	70.0%	65.0%

***Note:*** Grey text represents intervention coverage held constant from 2011–2016 during scenario; *Baseline ART and scale-up among adults based on county UNAIDS projections estimates.

The second set of scenarios focused on the impact of universal ART expansion alone in each country with equitable access for those female sex workers who are living with HIV and meet CD4 eligibility criteria. Equal access to ART services is a situation in which the proportion of female sex workers in need of ART, meeting CD4 eligibility criterion, and are receiving ART is comparable to the adult population in need and meeting eligibility that is also receiving ART. This scenario is followed by a scenario of universal ART expansion that is combined with the expansion of the community empowerment intervention for female sex workers. Table 2 describes baseline and 2016 target coverage for ART and the empowerment intervention for each country.

For each scenario, impacts are observed from 2012–2016 and compared to status quo, in which all interventions were held constant from 2011 levels forward. Results for each country are presented in figures as the annual number of new HIV infections among female sex workers. Recognizing that scenario 2 is both optimistic and feasible, we describe the cumulative infections averted among female sex workers and adults from 2012–2016 comparing the expanded intervention scenarios to status-quo coverage in the Results narrative.

## Results

**Figure 1:**
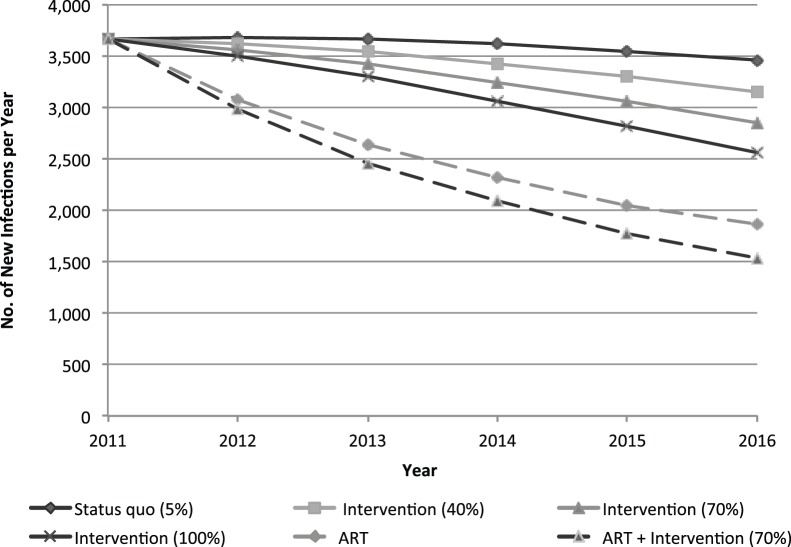
Annual new HIV infections among female sex workers in Brazil with expansion of the community empowerment-based HIV prevention intervention, with and without ART expansion among adults.

Across the four select countries, impacts vary as they are influenced by the background HIV prevalence among different risk groups and the overall adult population, risk behaviors, and population sizes. Figure 1 depicts the annual number of new infections among female sex workers per year in Brazil, according to each intervention and ART coverage scenario. Increasing intervention coverage to 70% among female sex workers in Brazil may avert a cumulative 1,830 HIV infections (10% reduction from status quo) among female sex workers and 4,740 infections among adults (3% reduction) from 2012–16, compared to status quo. Combined with equitable access to ART for those meeting criteria during expansion, the cumulative number of infections averted among female sex workers may reach 7,100 (40% reduction) over this five-year period.

**Figure 2:**
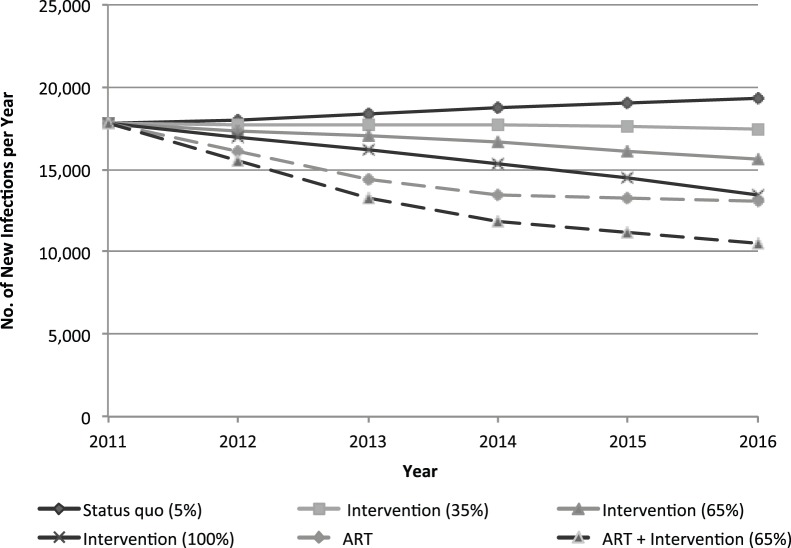
Annual new HIV infections among female sex workers in Kenya with expansion of the empowerment intervention, with and without ART expansion among adults.

Figure 2 depicts the annual number of new HIV infections among female sex workers per year in Kenya, according to each intervention and ART coverage scenario. Here, the expansion of the community empowerment intervention to 65% may avert a cumulative 10,800 infections among female sex workers (12% reduction) and may avert 20,680 adult infections (4% reduction) between 2012–2016. When the empowerment intervention is expanded simultaneously with equitable access to ART during expanded coverage of ART, 31,160 incident infections among female sex workers may be averted (33% reduction).

**Figure 3:**
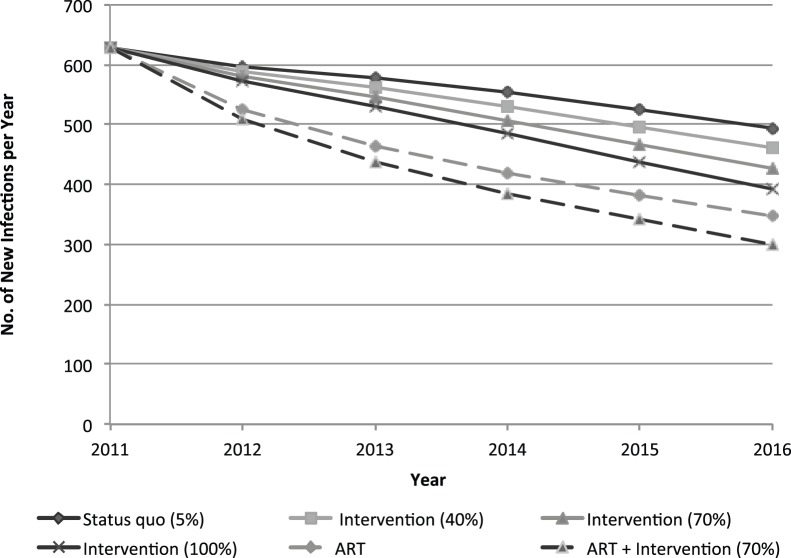
Annual new HIV infections among female sex workers in Thailand with expansion of the community empowerment-based prevention intervention, with and without ART expansion among adults.

In Thailand, approximately 220 female sex worker (8% reduction) and 730 adult infections (1% reduction) may be cumulatively averted when the community empowerment intervention reaches 70% coverage. Combining the increased coverage of the empowerment intervention with equitable access for sex workers during expansion of ART in Thailand may cumulatively avert almost 800 infections among female sex workers (28% reduction) over this five-year period. Figure 3 displays the number of new infections among female sex workers in Thailand from 2012–2016.

**Figure 4:**
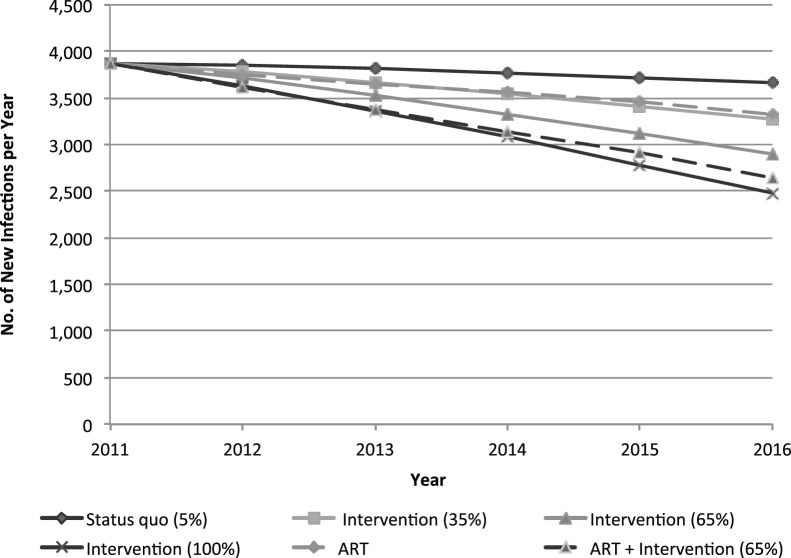
Annual new HIV infections among female sex workers in Ukraine with expansion of the community empowerment-based prevention intervention, with and without ART expansion among adults.

Figure 4 depicts the annual number of new HIV infections among female sex workers per year in Ukraine, according to each intervention and ART coverage scenario. Expansion of the community empowerment intervention to 65% may cumulatively avert 2,220 infections among female sex workers in Ukraine (12% reduction) and almost 7,000 infections among adults (3% reduction) from 2012 through 2016. The combined implementation of the empowerment intervention and equitable access to expanded ART for those meeting criteria in Ukraine may avert approximately 3,100 infections (17% reduction) among female sex workers within five years.

## Discussion

Expanding comprehensive, community empowerment-based HIV prevention among female sex workers has demonstrable impact on the HIV epidemics among female sex workers. The intervention resulted in a wide range of impacts across epidemiologically, politically and socially heterogeneous countries, with the lowest cumulative number of infections averted among sex workers estimated at 220 in Thailand and as high as 10,800 infections over five years in Kenya. Benefits also extend to the adult population where the expansion of the community empowerment intervention for female sex workers alone may cumulatively avert as few as 730 infections in Thailand to as many as 20,700 infections among adults in Kenya over five years.

Impacts of community empowerment HIV prevention on the HIV epidemic among the sex worker population and adult populations are influenced by the size of the sex worker population, as well as the existing coverage levels of ongoing HIV prevention interventions and/or HIV prevalence among female sex workers and adults. Such impacts are evident in Kenya, where the background prevalence of HIV is high among adults as well as female sex workers, and may avert almost 10,800 infections among female sex workers and 20,700 adult infections in five years. Though theoretical, these results are informative and demonstrate the potential impacts of targeted, empowerment-based interventions on both prioritized key populations, as well as, the general adult population.

Relatively smaller effects of the empowerment-based intervention in countries with concentrated epidemics such as Ukraine, Thailand, and Brazil should not be taken lightly. The community empowerment intervention may allow synergies across interventions through referral systems that could reduce program costs, and may reduce or maintain STI infection and unintentional pregnancy at low levels. For Thailand and Brazil, these secondary benefits are particularly relevant, given high levels of existing coverage of interventions and condom use. The impact of these interventions does not imply that sex work interventions should not be high on the prevention agenda; rather, coverage of comprehensive, community empowerment-based HIV prevention interventions must be maintained, at the minimum. This is particularly important that resources for HIV prevention are moving toward ART as prevention in many contexts. In Eastern Europe Central Asian countries and locations where HIV risks may also be related to injecting drug use for some sex workers, community coordinated integration of harm reduction services, such as needle and syringe programs, in the empowerment-based interventions may provide additional impacts. [17], [57].

Equitable access to ART demonstrates impressive benefits as well, cumulatively averting almost 800–31,200 new infections among female sex workers when combined with the expansion of the community empowerment intervention. These findings may be an underestimate, given that ART is allocated based on CD4 count in the model and does not reflect current barriers to ART access. Should sex workers be given equitable access, the change in coverage of ART among those sex workers in need may be higher and may have greater impacts than projected here, which models the change from current to nationally projected coverage. Few data exist to estimate unmet need of ART among sex workers, though evidence suggests female sex workers and other key populations often bear the greatest burden of HIV also often face discrimination and stigma when accessing treatment.[19], [58]–[60] Yet, HIV prevention interventions may have a limited impact without equitable access to ART for those living with HIV due to potential onward transmission and because the same factors that challenge ART provision and access may also challenge provision and access to other effective interventions. Finally, the impacts of ART do not imply that ART should be viewed as the only option for HIV prevention; maintaining or improving coverage of other interventions not only prevent STIs but are critical for preventing HIV infection and avoiding the need for and significant costs of ART.

Scale-up of ART and equitable access should be based on a human rights framework. Settings where one may be forced to disclose their involvement in sex work as their involvement in sex work or where one is required to provide a urine sample free of illicit drug chemicals in order to access services, may present significant barriers in ensuring that sex workers and other key populations are able to access services.[61]–[65] Furthermore, conflicting laws and public health policies, such as criminalization of sex work coupled with targeted HIV prevention programs for female sex workers, create confusions and/or risk for health providers and may place sex workers at risk of arrest or violence when accessing HIV prevention programs. [66] Countries which have implemented the community-based empowerment intervention have addressed these structural issues to maximizing realization of health rights and access to services. Efforts have targeted promotion and protection of sex workers’ human and labor rights, inclusion and participation in politics, stigma mitigation and reduction, and police interventions. [18] Others have gone further to address other structural barriers such as banking systems [67] and recognize sex work as an official occupation, meriting the same occupational rights as other professions. [68] As the need for effective, acceptable HIV risk reduction for sex workers maintains its position as a global priority, implementing interventions based on a human rights framework and addressing structural barriers opens the space for full realization and access to effective HIV prevention interventions.

### Limitations

The results should be reviewed in light of a several limitations. First, a potential limitation of this study results from the use of a single female sex worker risk group. One single group may not explicitly reflect the heterogeneity of sex workers in terms of their behaviors, observed HIV prevalence and other characteristics. To address this limitation, we conducted pooled analyses when faced with multiple and varied estimates, and readers should thus take this into consideration when reviewing the results. Further, the quality of mathematical models always reflects the quality and availability of data that are used to build the models. As new data become available on population size, epidemiology of risk groups, and behaviors, models will need to be updated to project future intervention impacts as populations change. Finally, the Goals model allows for analysis of the direct impacts of each intervention. These interventions likely have synergistic effects, through referral methods and improvements in ART adherence. Thus, these projections may underestimate the impacts associated with synergies that may be associated with the combined approach.

In July 2013, the WHO released revised guidelines, which recommend an initiation at CD4≤500 cells/mm^3^, as well as ART for HIV prevention among serodiscordant couples, including sex workers. [69] At the time of this analysis, ART initiation was recommended at a CD4≤350 cells/mm^3^, [54] reflecting the use of CD4 initiation at this cutoff. At the national level, these changes to ART criteria may not be immediately integrated into strategies, thus we believe the estimations provided here will remain applicable for some time until countries can meet this change in criteria and meet coverage needs and sex workers in need of treatment have equal access. Further modeling may be used to determine how equitable access at earlier initiation (CD4≤500 cells/mm^3^) may impact female sex worker and the wider adult populations in the future.

## Conclusion

The results of this mathematical modeling provided here, coupled with demonstrated effect provided by a recent review of these interventions, [18] suggest that a comprehensive package of interventions which supports a community empowerment approach to HIV prevention and access to ART for female sex workers may provide substantial impacts on the HIV epidemic among sex workers. Intervention impacts may vary according demographics, HIV epidemiology among adults and sex workers, as well as other social contexts in each country. These interventions, however, are a promising rights-based solution to overcoming the persistent burden of HIV among female sex workers across concentrated and generalized epidemics. Community-led initiatives may allow for more acceptable and sustainable programs and positive impacts on the HIV epidemics among female sex workers.
